# Cross-Linked Polymer Brushes Containing *N*-Halamine Groups for Antibacterial Surface Applications

**DOI:** 10.3390/polym13081269

**Published:** 2021-04-14

**Authors:** Selin Kinali-Demirci

**Affiliations:** 1 Department of Chemistry, Amasya University, Ipekkoy, 05100 Amasya, Turkey; selin.kdemirci@amasya.edu.tr; 2 Department of Biotechnology, Amasya University, Ipekkoy, 05100 Amasya, Turkey

**Keywords:** antibacterial surface, brush-gels, cross-linked polymer brushes, *N*-halamine

## Abstract

Microbial contamination is a significant issue in various areas, especially in the food industry. In this study, to overcome microbial contamination, cross-linked polymer brushes containing *N*-halamine were synthesized, characterized, and investigated for antibacterial properties. The cross-linked polymer brushes with different *N*-halamine ratios were synthesized by in-situ cross-linking methods with reversible addition−fragmentation chain transfer (RAFT) polymerization using a bifunctional cross-linker. The RAFT agent was immobilized on an amine-terminated silicon wafer surface and utilized in the surface-initiated RAFT polymerization of [*N*-(2-methyl-1-(4-methyl-2,5-dioxoimidazolidin-4-yl)propane-2-yl)acrylamide] (hydantoin acrylamide, HA), and *N*-(2-hydroxypropyl)methacrylamide) (HPMA) monomers. Measurement of film thickness, contact angle, and surface morphology of the resulting surfaces were used to confirm the structural characteristics of cross-linked polymer brushes. The chlorine content of the three different surfaces was determined to be approximately 8–31 × 10^13^ atoms/cm^2^. At the same time, it was also observed that the activation–deactivation efficiency decreased during the recharge–discharge cycles. However, it was determined that the prepared *N*-halamine-containing cross-linked polymer brushes inactivated approximately 96% of *Escherichia coli* and 91% of *Staphylococcus aureus*. In conclusion, in the framework of this study, high-performance brush gels were produced that can be used on antibacterial surfaces.

## 1. Introduction

Today, the need for various antimicrobial agents is increasing, especially coatings to prevent microorganism-related diseases. Most existing foodborne diseases are caused by foods contaminated with harmful microorganisms such as bacteria, yeast, viruses, etc. [1,2,3]. In the food industry, the loss of brand value and financial losses caused by microbiological contamination are the major problems that may be encountered [4,5]. For this reason, the need for antimicrobial agents that will effectively inhibit the growth of microorganisms at every step from production to the marketing of foods is increasing day by day, and new strategies to prevent microbial contamination are needed.

To prevent microbial contamination, many techniques have been developed in recent years [6,7,8,9,10]. For example, antibacterial surfaces based on an adenine-thymine base pairing are noteworthy for their reusability as well as for their relatively easy and fast preparations [11]. Another example is surfaces coated with polymeric ionic liquid. In particular, the hydrophobicity of polymeric ionic liquids can be controlled by the exchange of the counter ion [12]. In addition to the antibacterial properties of the prepared surfaces, this is also very useful in order to remove dead bacteria from surfaces. Other case studies reported the use of *N*-halamine derivatives to coat surfaces in order to confer them with antibacterial properties [13,14,15]. All of these methods point to that *N*-halamine could be very promising due to their reusability and their protective action. Furthermore, other features that support the use of *N*-halamines are strong antibacterial abilities, reactive ability, long-term stability, and low toxicity [15,16,17].

*N*-halamine-added polymeric materials have been successfully used in many different areas, and they are used in many studies, particularly to prevent foodborne diseases [18]. For example, Ma et al. developed an antibacterial polymeric coating containing halamine and dopamine groups [19]. The study makes the development of a product that can cover the surfaces of equipment used in food production quickly, cheaply, and with high performance possible. When existing foodborne diseases are considered, it is necessary to emphasize once again the importance of taking measures to prevent microorganism-related diseases at every step from production to consumption. In another study, Sun et al. developed a polymeric film containing *N*-halamine that can be used as food packaging [20]. It was determined that the prepared film was transparent, had good mechanical strength, and had high hydrophilic and biocidal properties. Furthermore, studies on antibacterial fabrics modified with polymers containing *N*-halamine have been included in many reviews [15,21,22]. However, studies on polymer brushes containing *N*-halamine are quite limited [23].

The polymer brushes can be defined as the layers formed by the terminated polymers where the polymer chains are bonded from one end to the solid surface [24,25]. Polymer brushes have found usage in many areas because they carry the desired functional groups and behave smartly [25,26,27]. Although various polymerization techniques can be used in the synthesis of polymer brushes, reversible-deactivation radical polymerization techniques have been used in many studies due to their advantages in recent years [28,29,30,31]. Thanks to these techniques, it has become possible to control the polymer’s molecular architecture and molecular weight in order to be synthesized and synthesize polymers with low polydispersity [32]. Among the reversible-deactivation radical polymerization techniques, atom transfer radical polymerization (ATRP), nitroxide-mediated polymerization (NMP), and reversible addition-fragmentation chain transfer (RAFT) polymerization are the most studied [32,33,34,35,36].

In addition to the unique properties of polymer brushes, their biggest disadvantage is that all or some of the polymer chains are removed from the surface over time, depending on environmental conditions [37]. To prevent this, cross-linked polymer brushes (brush gels) have started to be developed. Brush-gels have a unique structure as they combine the properties of polymer brushes and gels [38]. Studies have shown that cross-linked polymer brushes’ swelling properties can be controlled by the cross-link ratio, similar to gels. Furthermore, surface morphologies can likewise be controlled by the crosslinker ratio [39]. In this study, cross-linked polymer brushes containing *N*-halamine were synthesized on silicon wafers using RAFT polymerization. After the characterization of the synthesized brush gels, their antibacterial properties were also investigated. This new antibacterial material has a potential to be used in the development of high-performance and low-cost, easily applicable coatings, especially for food-related environmental surfaces.

## 2. Experimental

### 2.1. Materials

*N*-(1,1-Dimethyl-3-oxobutyl) acrylamide (≥99.0%, Sigma-Aldrich, Saint Louis, MO, USA), *N*-(2-hydroxypropyl)methacrylamide) (HPMA, 99.0%, Sigma-Aldrich), *N,N′*-methylenebisacrylamide (Bis-acrylamide, 99.0%, Sigma-Aldrich, Saint Louis, MO, USA), 4-cyano-4-[(dodecylsulfanylthiocarbonyl)sulfanyl]pentanoic (97.0%, Aldrich), 4,4′-azobis(4-cyanovaleric acid) (ACVA, ≥98.0%, Aldrich), ethyl alcohol (≥99.5%, Sigma-Aldrich), hydrogen peroxide (30 wt% in water, Sigma-Aldrich), potassium cyanide (≥97%, Sigma-Aldrich), ammonium carbonate (Sigma-Aldrich), hydrochloric acid (37%, Sigma-Aldrich), sulfuric acid (95.0–98.0%, Sigma-Aldrich), acetone (≥99.8%, Sigma-Aldrich), and *N*,*N*-dimethylformamide (DMF, 99.8%, Sigma-Aldrich) were used as received without further purification. Silicon (100) wafers (single side polished, N-type) were purchased from Aldrich. Deionized water (18.2 MΩ·cm) was obtained from a Milli-Q water purification system (Millipore, Bedford, MA, USA).

### 2.2. Synthesis of Halamine Vinyl Monomer (HA)

*N*-(1,1-Dimethyl-3-oxobutyl) acrylamide (5.0 mmol, 0.85 g), potassium cyanide (10.0 mmol, 0.65 g), and ammonium carbonate (30.0 mmol, 2.88 g) were stirred in 40.0 mL water/ethanol (1:1 by volume) solvent mixture at room temperature for 4 days. After removal of ethanol, diluted HCl was added to the crude product, and white solid [*N*-(2-methyl-1-(4-methyl-2,5-dioxoimidazolidin-4-yl)propane-2-yl)acrylamide] (hydantoin acrylamide, HA) was obtained after filtration. Yield: 85.6%. ^1^H NMR (600 MHz, DMSO-*d*_6_, δ, ppm): 1.23 (3H), 1.26 (3H), 1.30 (3H), 2.15 (2H), 5.48 (1H), 6.02 (1H), 6.25 (1H), 7.57 (1H), 7.83 (1H), 10.58 (1H). FTIR (ATR) *ν* (cm^−1^): 3217, 3070, 1772, 1729, 1707, 1664, 1635, 1542, 1454, 1277. Its physical and spectroscopic properties were compatible with the literature [19,40].

### 2.3. Surface-Initiated Polymerization and Activation of N-Halamine Precursor

**Scheme 1:**
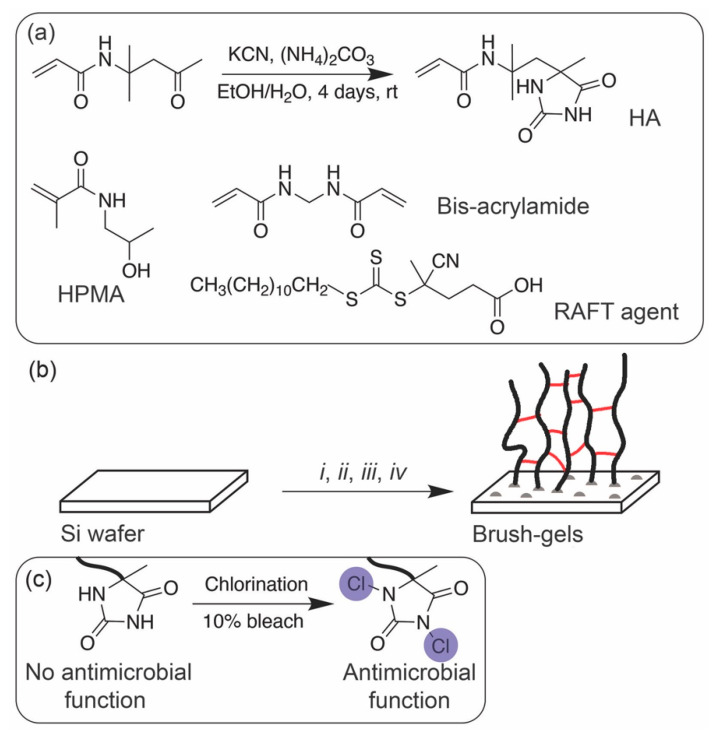
(**a**) Synthesis of hydantoin acrylamide (HA), and chemical structure of *N*-(2-hydroxypropyl)methacrylamide) (HPMA), Bis-acrylamide, and RAFT agent. (**b**) Preparation of brush-gels. *i*. Piranha solution, 2% HF. *ii*. t-BOC allylamine—UV, 25% TFA, 10% NH_4_OH. *iii*. RAFT agent. *iv.* HA, HPMA, cross-linker, RAFT agent, and initiator, 70 °C, 12 h. (**c**) Chlorination of *N*-halamine group.

Cross-linked polymer brushes were prepared by RAFT polymerization. The silicon wafers were initially cleaned in a “piranha” solution, which is a 3:1 mixture of concentrated H_2_SO_4_ and H_2_O_2_ (30%) heated to 90 °C for 30 min, followed by copious rinsing with deionized water. The cleaned silicon wafers were etched for 1 min in a 2% hydrofluoric acid solution, quickly rinsed in degassed deionized water, and dried in a stream of nitrogen. The allylamine molecule was bound by UV on the freshly prepared Si-H surfaces. The amine-modified silicon wafers were introduced into the solution of 4-cyano-4-[(dodecylsulfanylthiocarbonyl) sulfanyl] pentanoic acid in 20 mL of anhydrous dichloromethane. The preparation of the surfaces is shown in Scheme 1. RAFT agent-immobilized surfaces were placed in the glass reactor. *N*-(2-Hydroxypropyl)methacrylamide) (HPMA), *N*-halamine monomer (hydantoin acrylamide, HA), *N*,*N*′-methylenebisacrylamide as cross-linker, 4-cyano-4-[(dodecylsulfanylthiocarbonyl) sulfanyl] pentanoic acid as RAFT agent, 4,4′-azobis(4-cyanovaleric acid) (ACVA), (*Monomer*/*RAFT agent*/*Initiator*: 400/2/1), and 10 mL of DMF were added to a dry Schlenk tube and degassed three times. The solution was transferred to the glass reactor just before heating the reactor to 70 °C for 4 h to allow for polymerization, and cross-linked polymer brushes were obtained. The modified silicone surfaces were washed with DMF and acetone and dried with nitrogen gas.

To activate the *N*-halamine groups, the prepared surfaces were kept in 10% bleach solution for 30 min, washed with distilled water to remove free chlorine, and kept in the fume hood overnight. XPS and water contact angle measurements characterized crosslinked polymer brushes containing active *N*-halamine groups.

### 2.4. Antibacterial Surface

Antibacterial properties of the prepared surfaces were carried out by the colony-counting method [41]. Gram-negative *Escherichia coli* (ATCC 25922) and Gram-positive *Staphylococcus aureus* (ATCC 25923) strains were used in this study. Microorganisms were grown in 50 mL nutrient broth at 37 °C for 24 h. Briefly, 1 cm^2^ silicon surface sterilized with UV and bacteria were added to 10 mL nutrient broth. The bacterial suspensions’ turbidity was adjusted to a concentration of approximately 10^6^ cells/mL to meet 0.5 McFarland turbidity standards. After 24 h, colonies were counted.

The spread plate technique on a standard surface was used to count viable cells. All samples were serially diluted in 10 tubes in Ringer’s solution and spread on agar plates located on the shaker. Plates were incubated at 37 °C for 24 h, and colonies were counted. Results were calculated as average colony-forming units/mL. All experiments were done in triplicate and given as mean ± standard deviation.

### 2.5. Characterization

Fourier transform infrared (FTIR) spectra were recorded on a Nicolet 6700 instrument (Thermo Scientific, Waltham, MA, USA) with a Smart Orbit attenuated total reflection attachment. The spectra were taken at a resolution of 4 cm^−1^ after a 128-scan accumulation for an acceptable signal/noise ratio. The chemical composition information of the samples was obtained by X-ray photoelectron spectroscopy (XPS); the measurement was carried out on a Thermo Scientific K-Alpha spectrometer (Thermo Scientific, Waltham, MA, USA) using a monochromatic Al K-α X-ray source (*h*ν = 1486.6 eV). Charging neutralizing equipment was used to compensate sample charging, and the binding scale was referenced to the aliphatic component of C 1s spectra at 285.0 eV. The water contact angle measurements were conducted at room temperature using a goniometer (model DSA100, Krüss, Hamburg, Germany) equipped with a microliter syringe. Deionized water (5.0 μL) was used as the wetting liquid. The morphology of the silicon wafers was recorded on a Park Systems XE70 SPM Controller LSF-100 HS atomic force microscopy (AFM, South Korea). A triangular-shaped Si_3_N_4_ cantilever with integrated tips (Olympus, Tokyo, Japan) was used to acquire the images in the non-contact mode. The normal spring constant of the cantilever was 0.02 N/m. The force between the tip and the sample was 0.87 nN. The ellipsometric measurements were performed under ambient conditions using an ellipsometer (model EL X-02C, DRE Dr. Riss Ellipsometerbau GmbH, Ratzeburg, Germany) equipped with a He-Ne laser (λ = 632.8 nm) at a constant incident angle of 75°. The average dry thickness of polymer brushes on silicon substrate was determined by fitting the data with a three-layer model (native silicon (refractive index, *n* = 3.86) + silicon oxide layer (*n* = 1.46) + organic layer (*n* = 1.47) [37,39].

## 3. Results and Discussions

This study aimed to investigate the antibacterial properties of brush gels with different *N*-halamine ratios. The cross-linked polymer brushes containing *N*-halamine were synthesized by in-situ cross-linking methods with RAFT polymerization using a bifunctional cross-linker. The synthesis of HA, the surface modification, and the chlorination of *N*-halamine groups are schematically presented in Scheme 1. In addition to brush gels containing *N*-halamine group (**CB-25**, **50** and **75**), cross-linked polymer brushes without *N*-halamine (**CB-0**) were prepared. The cross-linker ratio was chosen to be 1%, and three different *N*-halamine ratios were used. For the sake of clarity, cross-linked polymer brush layers were named “**CB**” with variable HA ratios (**0**, **25**, **50**, and **75** for 0, 25, 50, and 75 mol % of HA in the monomer mixture, respectively).

**Figure 1:**
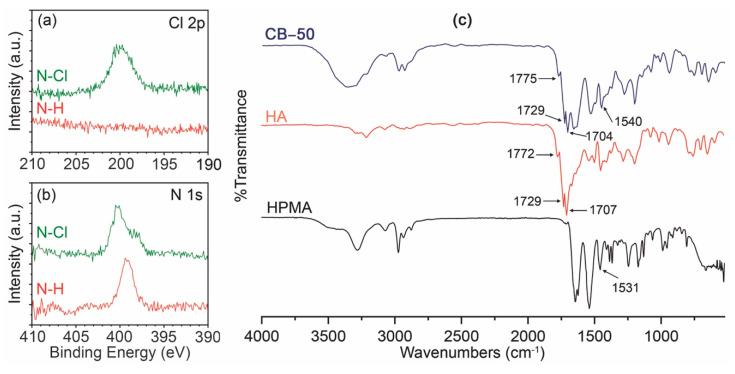
High-resolution XPS spectra of Cl 2p (**a**) and N 1s (**b**) spectra of **CB-50**. FTIR spectra of *N*-(2-hydroxypropyl)methacrylamide) (HPMA), hydantoin acrylamide (HA), and **CB-50** (**c**).

The chemical composition of the prepared surfaces was determined using XPS. The chemical compositions of the surfaces prepared after RAFT polymerization are summarized in Table 1. When the chemical compositions of cross-linked polymer brushes were determined, O 1s, N 1s, C 1s, and S 2p were four intensive elements in the main composition of all cross-linked polymer brushes. The presence of the C 1s (285 eV) and N 1s (400 eV, Figure 1a) provided evidence for the polymer layers. Besides that, no Cl 2p peak was observed (Figure 1b). Furthermore, it was determined that the Si 2s peak disappeared after polymerization due to the increasing surface thickness with polymerization. The board peak was observed in the 3400–3200 cm^−1^ region (O-H stretching vibration of HPMA). The characteristic bands of *N*-halamine monomer were observed at 3217, 1772, 1729, and 1707 cm^−1^ due to the N-H, and C = O stretching vibrations of the hydantoin groups (Figure 1c). The determination of the presence of characteristic peaks in the cross-linked polymer brushes (**CB-50**) shows that polymer brushes were properly synthesized.

**Figure 2:**
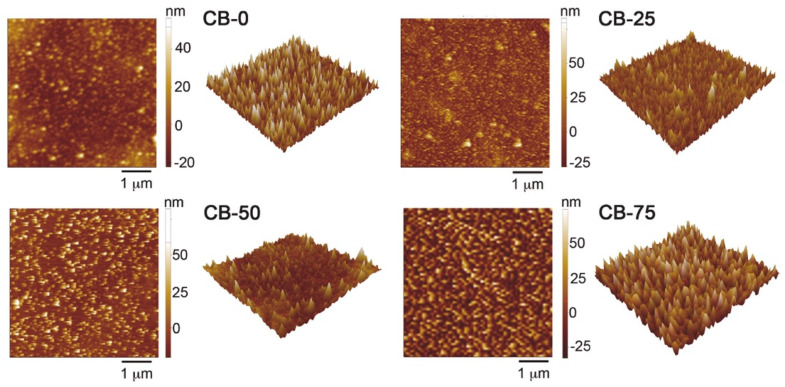
2D and 3D surface topography of **CB-0**, **CB-25**, **CB-50**, and **CB-75**.

The surface morphology of brush-gels was determined using AFM, as depicted in Figure 2. It was determined that the film morphologies appeared as needle-like structure heterogeneously distributed over the entire substrate area, where the root-mean-square (RMS) roughness of **CB-0**, **CB-25**, **CB-50**, and **CB-75** brush gels was 8.310, 7.870, 11.647, and 11.785 nm, respectively. The increase in RMS roughness values of the polymer brushes was possibly due to the increasing steric hindrance with the HA ratio.

**Figure 3:**
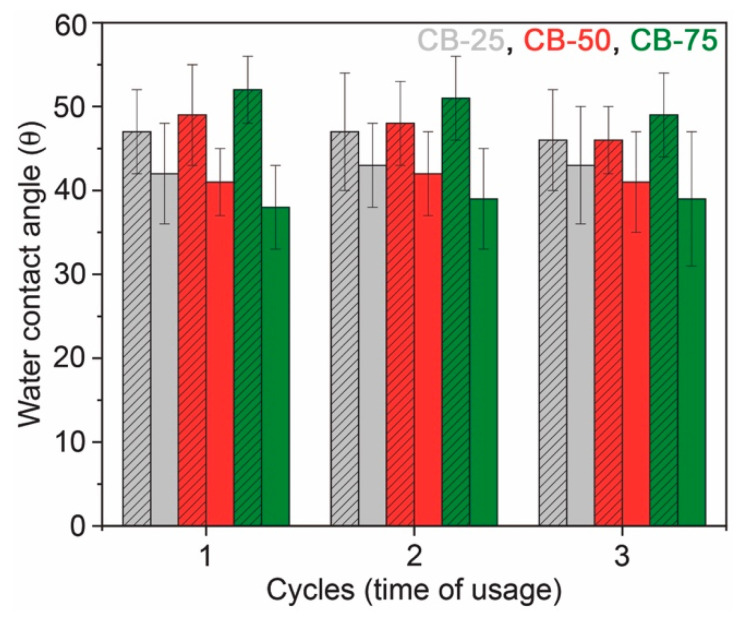
Water contact angles of and N-H (patterned) and N-Cl films of **CB-25**, **CB-50**, and **CB-75** in repeated cycles.

The hydrophobic and hydrophilic characteristics of brush gels were determined using water contact angle measurements. While the contact angle of the RAFT agent bonded surface was 65° [42], the brush gels’ water contact angle after polymerization was determined to be 42 ± 3°, 47 ± 5°, 49 ± 6°, and 52 ± 4° for **CB-0**, **CB-25**, **CB-50**, and **CB-75**, respectively. After strong chlorination, the water contact angle of cross-linked polymer brushes containing *N*-halamine significantly decreased to approximately 39 ± 4°; however, no significant change was observed in the water contact angle measurements of the *N*-halamine-free control surface (**CB-0**). Water contact angle measurements were repeated over three recharge–discharge cycles to investigate the reusability of the surfaces, and the cross-linked polymer brushes can still be reused after recharge (Figure 3).

**Figure 4:**
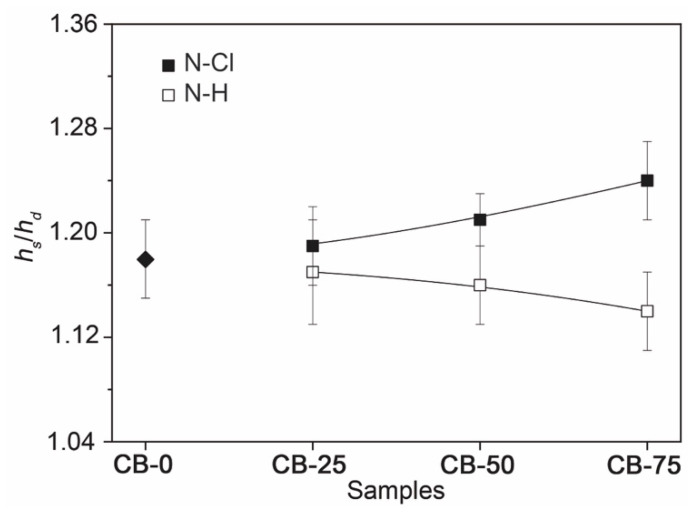
Swelling ratio *h_s_*/*h_d_* of **CB-0**, **CB-25**, **CB-50**, and **CB-75**.

After each cycle, the ellipsometric thickness of the brush gels was determined in addition to hysteresis measurements. Hysteresis values are a measure of the surface roughness and increase with increasing surface roughness. It was determined that the hysteresis values slightly increased from 12 ± 3° to 17 ± 5° after the activation–deactivation processes. This was proven by the ellipsometric thickness of the surfaces. The ellipsometric thickness decreased by about 12% after three recharge–discharge cycles because of the leaving groups. The swelling ratios of the prepared surfaces were calculated by measuring their wet (*h_s_*) and dry (*h_d_*) thicknesses. The swelling ratio (*h_s_*/*h_d_*) is a function of grafting density and cross-linked ratio. However, in this study, the cross-linker ratio was kept constant. So, the differences between the swelling ratios of **CB** surfaces come from hydrophilicity and the intermolecular hydrogen bond. The swelling ratio of *N*-halamine precursor was slightly decreased compared with **CB-0** due to the decreasing hydrophilicity. On the other hand, the swelling ratio of N-Cl films was increased with increasing hydrophilicity. **CB-75**, which contains 75% of *N*-halamine monomer, showed a higher swelling ratio (Figure 4). The increase in swelling ratio of the polymer brushes was possibly due to the increasing hydrophilicity with HA ratio.

**Figure 5:**
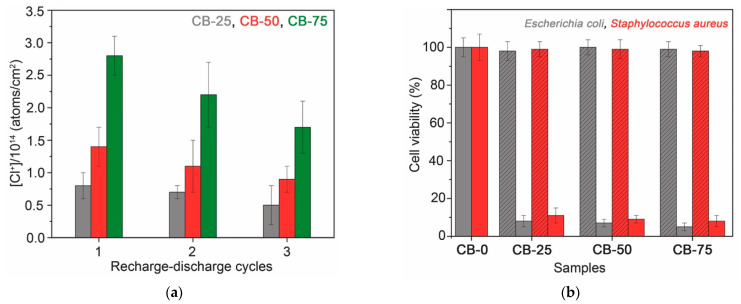
(**a**) The oxidative chlorine content of **CB-25**, **CB-50**, and **CB-75** after different “recharge−discharge” cycles. (**b**) Cell viability (%) of *Escherichia coli* and *Staphylococcus aureus* colonies treated by N-H (patterned) and N-Cl films of **CB-0**, **CB-25**, **CB-50**, and **CB-75**.

The activation of the *N*-halamine-containing surfaces was also proven by the XPS results. After activation with bleach, it was observed that the N 1s peak shifted to 400 eV, and its shape changed after activation (Figure 1a). Furthermore, the Cl 2p peak was observed at 200 eV (Figure 1b). This suggests that the N-H bond was transformed into the N-Cl bond. The amount of oxidative chlorine on the surface after activation was determined using titration. The chlorine content for the three different surfaces was determined to be approximately 8–31 × 10^13^ atoms/cm^2^. At the end of the activation–deactivation processes, it was determined that the chlorine content of the surfaces decreased by about 35% (Figure 5a). This can be explained by the decrease in the activation–deactivation efficiency of cross-linked polymer brushes in accordance with the water contact angle and hysteresis measurements.

In this study, the antibacterial properties of cross-linked polymer brushes containing *N*-halamine were determined using the colony count method with Gram-positive *Escherichia coli* and Gram-negative *Staphylococcus aureus* bacteria. No significant change was observed in the viable cell counts of deactivated (N-H) cross-linked polymer brushes in compared with the control surface (**CB-0**). However, it was determined that after the activation of the surfaces with bleach, the cell viability was decreased for **CB-25**, -**50**, and -**75**, almost 4% for *Escherichia coli* and 9% for *Staphylococcus aureus* were live (Figure 5b). As the number of recharge−discharge cycles of the prepared surfaces increased, no significant change was observed in the antibacterial properties of the brush gels with a high *N*-halamine ratio.

## 4. Conclusions

In this study, cross-linked polymer brushes containing *N*-halamine were synthesized on silicon wafers using RAFT polymerization. The characterization of the synthesized brush gels was carried out with XPS, FTIR, AFM, water contact angle, and ellipsometer measurements. It was observed that the swelling ratio increased with the increasing HA ratio. The chlorine content for the three different surfaces was determined to be approximately 8–31 × 10^13^ atoms/cm^2^. After recharge–discharge cycles, it was determined that the chlorine content of the surfaces decreased by about 35%. The antibacterial test showed that approximately 94% of the *Escherichia coli* and 91% of the *Staphylococcus aureus* bacteria that came into contact with the active cross-linked polymer brushes were killed. In conclusion, the multi-directional cross-linked polymer brushes containing *N*-halamine developed in this study have great potential for antibacterial surface applications in many different fields, especially in the food industry.

## Figures and Tables

**Table 1:** Atomic concentration brush-gel layers as determined by XPS analysis ^1^.

Entry		O 1s	N 1s	C 1s				Cl 2p	S 2p	Si 2p
				N-C = O	C-O	C-N	C-C			
**Si-NH_2_**	Energy (eV) Conc. (%)	532.2 13.2	400.0 16.1	– 52.8	–	285.6	285.0	– –	– –	99.1 17.9
**Si-RAFT**	Energy (eV) Conc. (%)	532.2 21.5	399.8 8.3	288.6 52.6	–	286.2	285.0	– –	163.1 9.3	99.1 8.3
**CB-0**	Energy (eV) Conc. (%)	532.5 22.7	400.1 8.9	288.9 66.8	286.8	285.7	285.0	– –	163.1 1.6	– –
**CB-25 ^2^**	Energy (eV) Conc. (%)	532.3 22.6	399.8 10.1	288.7 65.9	286.7	285.6	285.0	– –	163.2 1.4	– –
**CB-50 ^2^**	Energy (eV) Conc. (%)	532.2 21.4	399.9 11.4	288.7 66.3	286.8	285.7	285.0	– –	163.1 0.9	– –
**CB-75 ^2^**	Energy (eV) Conc. (%)	532.1 20.8	399.8 13.7	289.0 64.8	286.8	285.5	285.0	– –	162.9 0.7	– –

^1^ Binding energies were calibrated to aliphatic carbon at 285 eV. ^2^ *N*-Halamine precursor (N-H).
